# The cognitive consequences of the COVID-19 infection and behavioral variant frontotemporal dementia: Is there a link?

**DOI:** 10.1192/j.eurpsy.2023.1671

**Published:** 2023-07-19

**Authors:** G. I. Costandache, B. A. Oroian, A. P. Salaru, P. F. Ionescu, C. Mihai

**Affiliations:** 1 Resident Psychiatrist, Institute of Psychiatry Socola; 2 Associate Professor, University “Apollonia”; 3 Md, Ph.d, Institute of Psychiatry Socola, Iasi, Romania

## Abstract

**Introduction:**

Frontotemporal dementia behavioral variant (bvFTD) is the most common subtype of frontotemporal dementia, characterized by early and often severely disabling alterations in personality and social conduct that carry a huge impact on the patient, family, and society.

**Objectives:**

The aim of the study was to correlate the clinical data collected from our patient with relevant literature and discuss the diagnosis of bvFTD in the context of the COVID-19 pandemic.

**Methods:**

Case report and a systematic review of the literature.

**Results:**

The middle-aged female patient we examined presented an array of psychiatric symptoms, including cognitive, behavioral, and personality changes that emerged in two months after a mild form of SARS-CoV-2 infection. Objectively, a cranial CT scan displayed frontal and anterior temporal lobe atrophy. The rapid and severe decline of the patient’s mental faculties throughout the last year, along with the circumstances in which the pathology developed, raised a question about the etiological factors that contributed to this early-onset dementia.

**Conclusions:**

Although diagnostic criteria are useful, frontotemporal dementia may be difficult to differentiate from other conditions because there are no disease-specific biomarkers. Correlations between the COVID-19 infection and the fulminant bvFTD symptoms remain unclear and require further investigations.

**Disclosure of Interest:**

None Declared

